# Precise identification of intersectional hybrids in *Morus* using genomic *in situ* hybridization (GISH)

**DOI:** 10.48130/forres-0026-0009

**Published:** 2026-04-03

**Authors:** Qiming Zhou, Jiacheng Li, Yahui Xuan, Jiali Qiu, Jianglian Yuan, Zhengang Li, Song Chen, Ningjia He

**Affiliations:** 1 State Key Laboratory of Resource Insects, Southwest University, Beibei, Chongqing 400715, China; 2 Sericulture and Apiculture Research Institute, Yunnan Academy of Agricultural Sciences, Mengzi, Yunnan 661101, China

**Keywords:** Mulberry, Intersectional hybrids, Hybrid identification, GISH, 2*n* gametes

## Abstract

Mulberry (*Morus* spp.) includes ecologically important tree species that are highly valued for their exceptional economic and medicinal properties. Among its diverse species, *Morus wittiorum* and *Morus laevigata* are particularly valuable genetic resources because of their resistance to *Sclerotinia*, their relatively high content of specific flavonoids, and elongated fruit morphology. In this study, hybridization experiments were conducted using 10 mulberry accessions spanning three taxonomic sections (*Alba*, *Wittiorum*, and *Laevigata*). All six attempted intersectional crosses successfully yielded hybrid progeny. Using genomic *in situ* hybridization with blocking DNA, we detected distinct chromosomal signal patterns among the three sections, enabling precise identification of hybrid and wild-type chromosomal constitutions. Notably, this study provides the first documented evidence of 2*n* gamete formation in the genus *Morus*, where 2*n* eggs from *M. wittiorum* 'W-4' produced a pentaploid hybrid, 'Mp-7'. This discovery not only rewrites the chromosomal inheritance patterns of *Morus* but also unveils untapped polyploid breeding potential. These findings provide an efficient approach for identifying hybrids and offer novel polyploid breeding strategies, thereby promising to reshape global mulberry breeding and creating new opportunities for the genetic improvement of this agronomically important species.

## Introduction

Mulberry (*Morus* spp.), a perennial woody plant belonging to the family Moraceae in the order Rosales^[1]^, is not only the primary food source for silkworms (*Bombyx mori*) but also possesses significant medicinal value, including blood glucose regulation and antioxidant properties^[2,3]^. Hybridization serves as a key strategy in plant improvement, facilitating novel gene flow and enhancing desirable traits across diverse plant species^[4−6]^. In mulberry breeding, hybrids exhibiting potential salt tolerance have been developed^[7]^. Early hybrid identification is crucial for enhancing breeding efficiency. However, studies on the authentication of *Morus* hybrids remain limited. Traditional identification methods, such as morphological trait observation^[8]^, are often inadequate because of the pronounced morphological variation observed in mulberry hybrids compared with their parental lines^[9]^. Therefore, relying solely on morphological characteristics for identifying hybrids is unreliable.

High-throughput molecular DNA markers provide a robust framework for hybrid screening. For instance, 11 single nucleotide polymorphism (SNP) markers have been used to distinguish intraspecific and interspecific hybrids of grain amaranth (*Amaranthus* spp.)^[10]^. Similarly, 70 simple sequence repeat (SSR) primers and 8 inter-simple sequence repeat (ISSR) primers have been used to detect genetic diversity and polymorphism in *Lilium*, facilitating the identification of hybrids and analyses of their genetic relationships^[11,12]^. In *Morus*, phylogenetic analysis using internal transcribed spacer and sequence-characterized amplified region markers has enabled the discrimination of putative interspecific hybrids^[9,13,14]^. However, molecular marker-based hybrid identification faces challenges in polyploid genomes, where the presence of polymorphic loci and varying ploidy levels complicates the correspondence between markers and species^[15,16]^. This limitation is particularly prominent in autopolyploid species; for instance, SNP markers often fail to differentiate between allelic variants and homologous SNPs arising from genome duplication^[17]^. The genus *Morus* exhibits extensive ploidy variation and frequent interspecific hybridization, resulting in a highly complex genetic background^[18−22]^. Consequently, though molecular DNA markers offer partial insights into hybrid identification, their efficacy is limited in dissecting the precise genomic contributions of the parental lineages.

Cytogenetics, particularly molecular cytogenetic tools such as fluorescence *in situ* hybridization and genomic *in situ* hybridization (GISH), is a powerful approach for identifying hybrids and genomic characterization in plants^[23]^. For instance, hybrid populations in the genus *Veronica* were successfully identified by comparing the signal numbers of 45S and 5S ribosomal DNA (rDNA) between parents and offspring^[24]^. Similarly, GISH analysis enabled the accurate identification of intergeneric hybrids between *Saccharum officinarum* and *Narenga porphyrocoma* while revealing their chromosomal constitution and *n* + *n* inheritance patterns^[25]^. Recently, the application of GISH has elucidated the genetic relationships among 12 species and three varieties in the genus *Morus*, leading to the establishment of a cytotaxonomy system comprising five sections and two subsections^[20]^. Subsequent studies employing comparative GISH (cGISH) with blocking DNA and self-GISH refined this system further, proposing the division of the section *Wittiorum* into two sections, *Wittiorum* and *Laevigata*. Notably, GISH offers several advantages, including reference-genome-free analysis, cost-effectiveness, rapid processing, and intuitive chromosomal visualization. These features make it particularly valuable for studying wild *Morus* species such as *Morus* *laevigata* and *Morus* *wittiorum*, which lack reference genomes. Unlike molecular markers (e.g., SSRs or SNPs) that provide fragmented genetic locus information, GISH enables the direct identification of parental chromosome sets and their recombination events in hybrid progenies^[26]^. Although GISH has limitations in detecting small-fragment genomic structural variations and in discriminating species with extremely close genetic relationships^[27]^, the technique remains critical for analyzing polyploid *Morus* hybrids, where comprehensive chromosomal insights are essential.

The formation of 2*n* gametes in plants can occur through pre-meiotic or postmeiotic genome doubling, as well as via meiotic restitution^[28−30]^. These gametes have been widely documented across diverse plant species, including wild potato (*Solanum malmeanum*) and lemon (*Citrus limon*)^[31−33]^. As a pivotal mechanism for sexual polyploidization, 2*n* gametes contribute significantly to spontaneous polyploid formation in plants^[29,34,35]^. For instance, the detection of 2*n* gametes in cultivated caladium (*Caladium* × *hortulanum*) and oil palm (*Elaeis guineensis*) has provided direct evidence for naturally occurring sexual polyploidization events in these species^[36,37]^. Furthermore, 2*n* gametes serve as a crucial tool for artificial polyploid induction, offering significant potential for plant breeding^[38]^. High-temperature treatment has been shown to induce unreduced 2*n* pollen, with successful applications reported in *Lagerstroemia indica* and *Camellia* *oleifera*^[39,40]^. However, no evidence for the occurrence of 2*n* gametes has been reported in the genus *Morus*, highlighting a critical gap in polyploidy research and mulberry breeding programs.

The exceptional resistance of *M. laevigata* to *Sclerotinia scleroterum* and its elongated fruit phenotype underscore its potential as valuable genetic resources for introgression via hybridization^[41]^. Moreover, *M. wittiorum* 'Twcgs' was found to contain significantly higher levels of rutin and nicotiflorin compared with other mulberry cultivars^[42]^. Previous studies have also shown that hybridization can effectively enhance the content of quercetin 3-(6-malonylglucoside)^[43]^. On the basis of these findings, six cross combinations were designed as part of breeding programs aimed at improving fruit length, enhancing resistance to *S*. *scleroterum*, and increasing flavonoid accumulation. In this study, GISH was used to confirm the hybrid authenticity of seven hybrid progenies derived from these six cross combinations and to characterize the chromosomal composition of two wild mulberry accessions. Comparative analysis against the respective parental lines conclusively verified the hybrid status of all seven progenies. This research provides a reliable method for early identification of mulberry hybrids, which facilitates the effective utilization of mulberry resources and supports advanced genetic breeding programs.

## Materials and methods

### Plant materials

Intersectional hybridization experiments were conducted using 10 mulberry accessions (*Morus* spp.) selected from three taxonomic sections (*Alba*, *Wittiorum*, and *Laevigata*). These accessions were selected because of their superior morphological traits and typical cytotaxonomic characteristics, prioritizing germplasm possessing homologous chromosomes^[20]^. All accessions, including the wild genotypes and hybrids, were maintained through grafting or seeding in the Mulberry Germplasm Nursery at Southwest University, Chongqing, China. Three representative accessions, namely *Morus multicaulis* 'Heyebai' (*Ma*), *M. laevigata* 'Menghai No. 2' (*Ml*), and *M. wittiorum* 'Ailaoshan No. 9' (*Mw*), were used for genomic DNA extraction. Detailed information regarding the mulberry accessions, including those used for genomic DNA extraction, the parental lines, and the wild specimens, is provided in Supplementary Table S1. Additionally, the seven hybrids from six cross combinations are documented in Supplementary Table S2.

### Chromosome preparation

Mitotic chromosome preparation followed established protocols^[44]^. Briefly, fresh leaf and root tip samples were pretreated with 2 mM 8-hydroxyquinoline at room temperature for 3 h, fixed in 3:1 (v/v) ethanol : glacial acetic acid for 4 h, and stored in 70% ethanol at 4 °C. After three washes with distilled water, the tissues were enzymatically digested at 37 °C in a solution containing 2% (w/v) cellulase Onozuka R-10 (YaKult, Japan) and 1% (w/v) pectolyase Y-23 (YaKult, Japan) (pH 5.5). The duration of digestion varied by tissue type: 3 h for leaves and 1 h for root tips. The digested samples were rinsed with 70% ethanol and macerated into a cell suspension. For slide preparation, one drop of the cell suspension was mixed with glacial acetic acid. Chromosome spreads were observed using an Olympus IX73 microscope (Olympus, Japan).

### Genomic probe labeling and preparation of blocking DNA

Genomic DNA was extracted from young leaves of *Ma*, *Ml*, and *Mw* using the DNAquick Plant System kit (TIANGEN BIOTECH, Beijing, China) according to the manufacturer's instructions. Genomic DNA was then fluorescently labeled by nick translation with either ChromaTide Alexa Fluor 488-5-dUTP (Thermo Fisher Scientific [Invitrogen], Massachusetts, USA) or Texas-Red-5-dCTP (PerkinElmer, Massachusetts, USA)^[20]^. Blocking DNA (~200-bp fragments) was generated by autoclaving genomic DNA from *Ma*, *Ml*, and *Mw* at 121 °C for 2 min.

### Genomic *in situ* hybridization

GISH analysis was performed according to our previously established protocol^[20]^. Chromosome slides were crosslinked by ultraviolet light at 1,250 mJ/cm^2^ for 2 min prior to hybridization. The probe mixture (15 ng/μL of fluorescently labeled DNA in a hybridization buffer: 2 × Saline-Sodium Citrate Buffer (SSC) and 1 × TE; pH 7.0) was denatured with chromosomes at 100 °C for 5 min and then incubated overnight at 42 °C. For GISH with blocking DNA, the hybridization mixture was supplemented with blocking DNA (30× the probe concentration). After being washed in 2× SSC at room temperature for 5 min, the slides were counterstained with 4',6-diamidino-2-phenylindole (DAPI) (1 ng/μL) and sealed with nail polish. For multiple rounds of GISH, the probes were stripped in 50% formamide (containing 2× SSC) at 42 °C for 10 min after removing the coverslips. The slides were dehydrated in an ethanol series (70%, 90%, and 100% for 3 min each at room temperature) before the next hybridization round. Images were captured using an Olympus DP80 CCD camera (Olympus, Japan) under an Olympus IX73 fluorescence microscope and processed using Adobe Photoshop 2021 and Adobe Illustrator 2021 (Adobe, USA).

To ensure accurate and reliable GISH signal quantification, signals were defined as discrete fluorescent foci exceeding an intensity threshold of 50 arbitary units (AU). For each hybrid individual, at least 20 metaphase cells exhibiting a clear chromosome distribution were imaged. Enumeration of the signals within these cells was independently performed by three researchers (Qiming Zhou, Jiacheng Li, and Ziang Li of Nanjing Agricultural University, Nanjing, China), with the results used for subsequent analyses.

## Results

### Production of intersectional hybrids

*M. wittiorum* and *M. laevigata* exhibit the longest fruit morphologies in the genus *Morus* (Supplementary Fig. S1), making them valuable genetic donors for intersectional hybridization programs focused on improving fruit traits. In the present study, 10 mulberry accessions spanning three taxonomic sections (*Alba*, *Wittiorum*, and *Laevigata*) were selected as parental lines for systematic intersectional crosses (Supplementary Table S1), with the crossing scheme detailed in Supplementary Table S2. All designed crosses successfully produced hybrid progeny. Crosses 1−6 yielded 280, 816, 5, 85, 36, and 925 seeds, respectively. For cytological verification of *the plants'* hybridity, one vigorous individual plant was randomly selected for each cross. To assess their chromosomal composition, GISH was performed using the representative cultivars *Ma* (*Alba*), *Ml* (*Laevigata*), and *Mw* (*Wittiorum*), as either genomic probes or blocking DNA (Supplementary Table S1). This analysis encompassed seven progeny lines derived from six crosses (Supplementary Table S2).

### GISH analysis of intersectional hybrids between *Morus* section *Alba* and section *Wittiorum*

Two intersectional hybrids were successfully generated between *Morus* section *Alba* and section *Wittiorum*: (1) *M. alba* 'A-1' × *M. wittiorum* 'W-1' and (2) *M. wittiorum* 'W-2' × *M. alba* 'A-2'. Genomic probes derived from *Ma* and *Mw* were used reciprocally as either hybridization probes or blocking DNA to analyze chromosomal compositions. An initial GISH analysis without blocking DNA revealed substantial signal co-localization between the *Ma* and *Mw* probes in both the parents and the hybrid progeny, preventing unambiguous chromosomal assignment (Supplementary Fig. S2). To resolve this limitation, cGISH was subsequently performed by using standardized blocking DNA, which enabled precise discrimination of the parental chromosomal contributions in the hybrid offspring.

**Figure 1:**
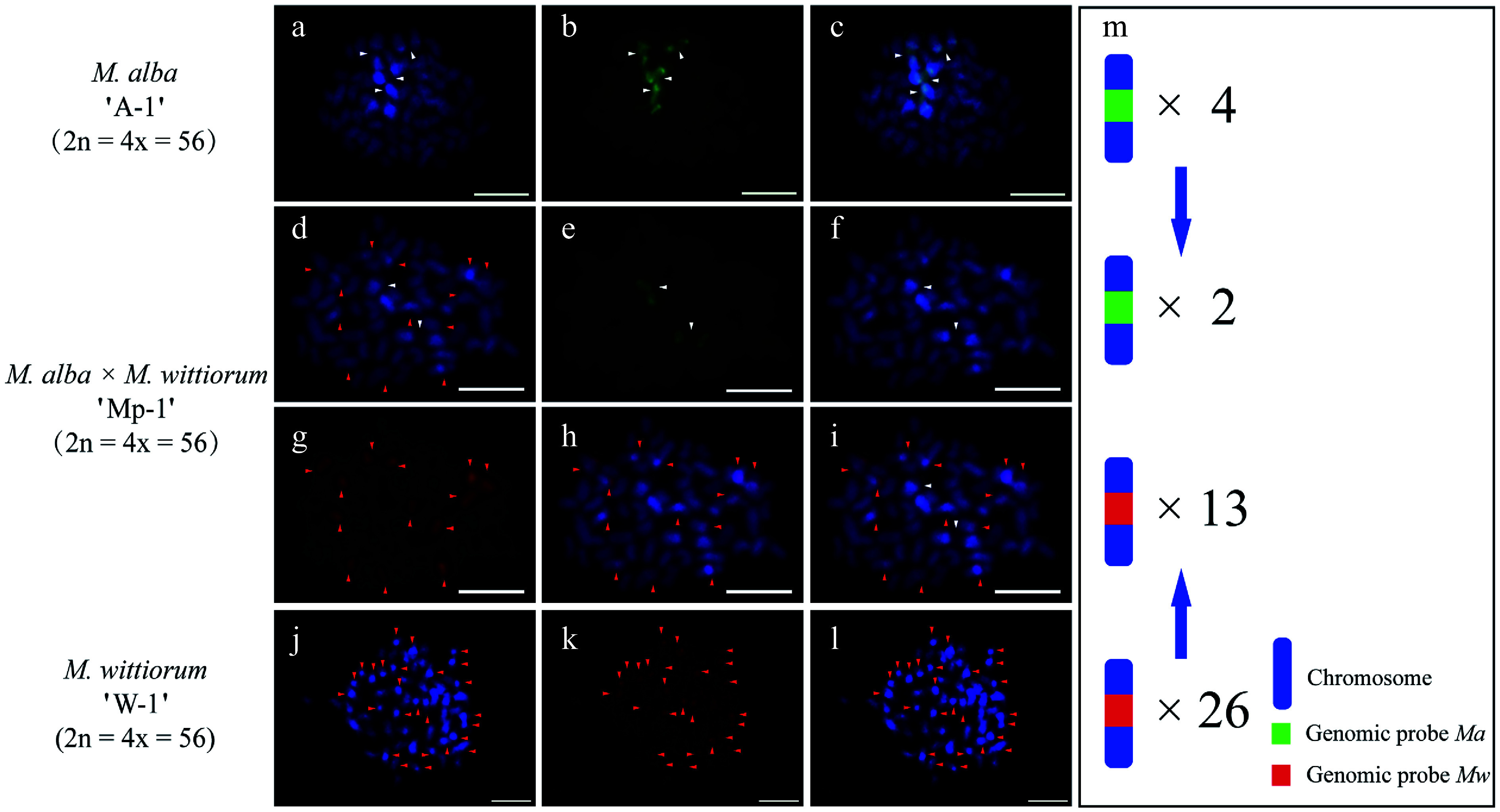
The cGISH signal patterns in *M. alba* 'A-1', *M. wittiorum* 'W-1', and their hybrid *M. alba × M. wittiorum* 'Mp-1'. cGISH signals using the genomic probe from *M. alba* (*Ma*) (green) with blocking DNA from *M. wittiorum* (*Mw*) were detected in (a)−(c) *M. alba* 'A-1' and (d)−(f) the hybrid 'Mp-1'. cGISH signals using the genomic probe from *Mw* (red) with blocking DNA from *Ma* were detected in (g), (h) the hybrid 'Mp-1' and (j)−(l) *M. wittiorum* 'W-1'. (i) The two-round cGISH showing overlapping signals from *Ma* (green) and *Mw* (red) genomic probes with reciprocal blocking DNA. (m) Ideogram summarizing chromosome counts with different genomic probes' signals. White and red arrows point to chromosomes with *Ma* and *Mw* signals, respectively. Blue arrows indicate transmission of the parental chromosome to the hybrid. Scale bars represent 5 μm.

**Figure 2:**
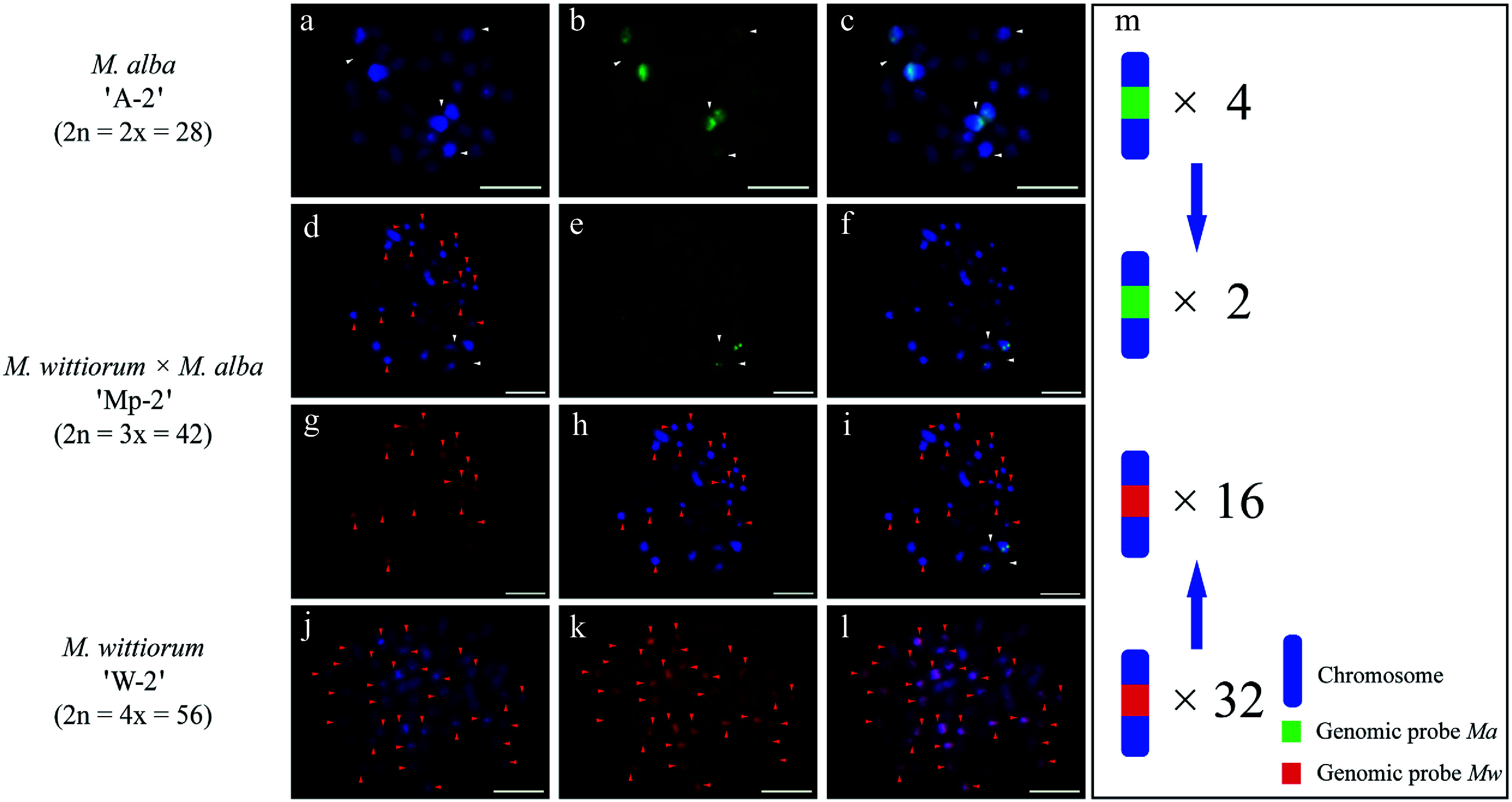
cGISH signal patterns in *M. alba* 'A-2', *M. wittiorum* 'W-2', and their hybrid *M. wittiorum × M. alba* 'Mp-2'. cGISH signals using the genomic probe from *Ma* (green) with blocking DNA from *Mw* were detected in (a)−(c) *M. alba* 'A-2' and (d)−(f) the hybrid 'Mp-2'. cGISH signals using the genomic probe from *Mw* (red) with blocking DNA from *Ma* were detected in (g), (h) the hybrid 'Mp-2' and (j)−(l) *M. wittiorum* 'W-2'. (i) The two-round cGISH showing overlapping signals from *Ma* (green) and *Mw* (red) genomic probes with reciprocal blocking DNA. (m) Ideogram summarizing the chromosome counts with different genomic probes' signals. White and red arrows point to the chromosomes with *Ma* and *Mw* signals, respectively. Blue arrows indicate the transmission of parental chromosome to the hybrid. Scale bars represent 5 μm.

In the *M. alba* 'A-1' × *M. wittiorum* 'W-1' cross, cGISH analysis revealed parental genomic distributions as follows. The *Ma* probe with *Mw* blocking DNA identified four hybridization signals in the female parent (Fig. 1a−c). The *Mw* probe with *Ma* blocking DNA detected 26 signals in the male parent (Fig. 1j−l). Their hybrid progeny, 'Mp-1', showed differential incorporation with two *Ma*-derived and 13 *Mw*-derived signals (Fig. 1d−i). The cross *M. wittiorum* 'W-2' × *M. alba* 'A-2' exhibited parallel findings. The *Mw* probe with *Ma* blocking revealed 32 signals in the female parent (Fig. 2j−l). The *Ma* probe with *Mw* blocking detected four signals in the male parent (Fig. 2a−c). The hybrid 'Mp-2' demonstrated two *Ma*-specific and 16 *Mw*-specific signals (Fig. 2d−i).

### GISH analysis of intersectional hybrids between *Morus* section *Alba* and section *Laevigata*

Consistent with the cross combinations of section *Alba* and section *Wittiorum*, genomic probes targeting *Ma* and *Ml* exhibited extensive signal overlap (Supplementary Fig. S3). This ambiguity required the application of cGISH with species-specific blocking DNA to resolve the genomic origins.

**Figure 3:**
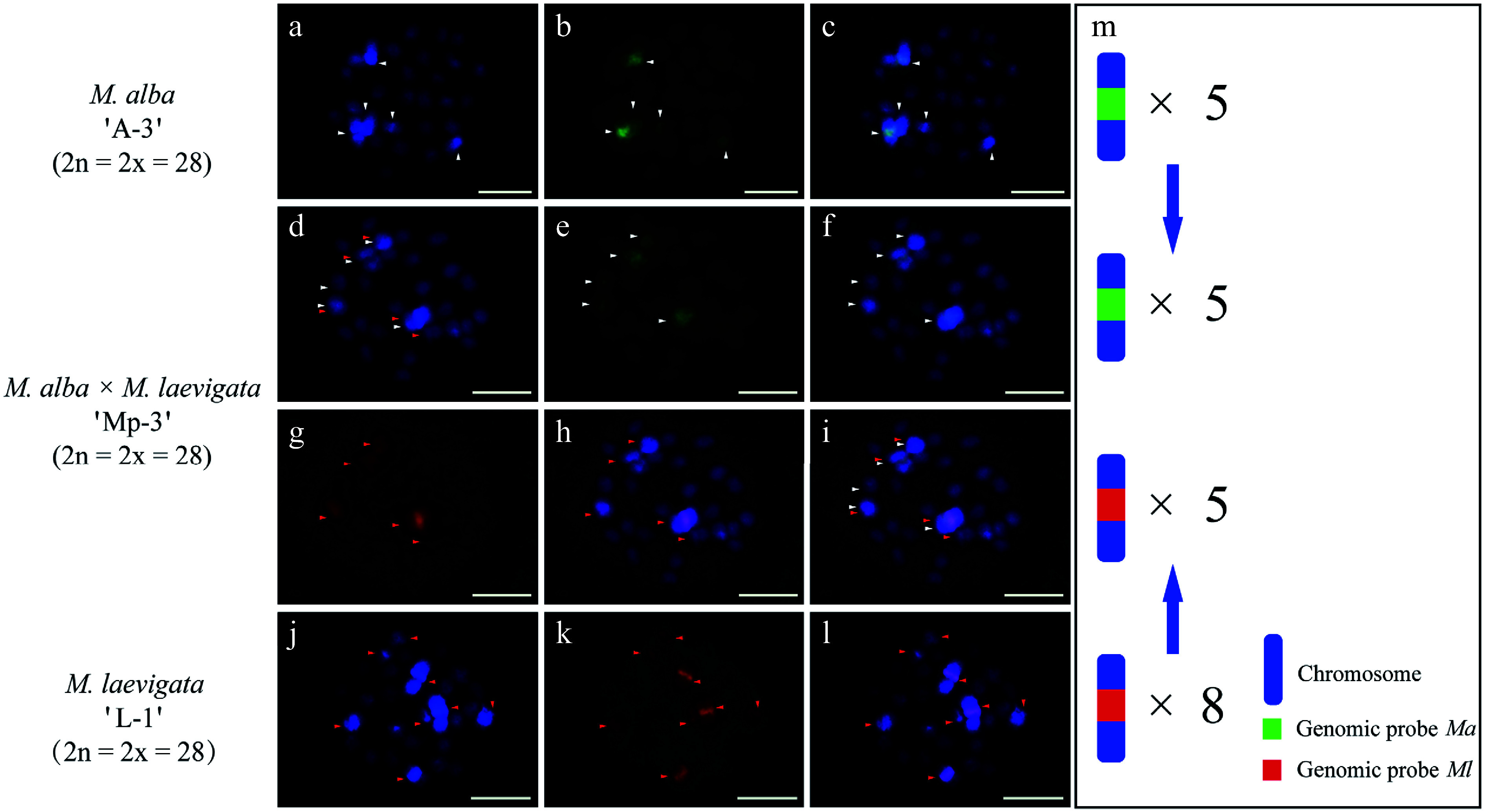
cGISH signal patterns in *M. alba* 'A-3', *M. laevigata* 'L-1', and their hybrid *M. alba × M. laevigata* 'Mp-3'. cGISH signals using the genomic probe from *Ma* (green) with blocking DNA from *M. laevigata* (*Ml*) were detected in (a)−(c) *M. alba* 'A-3' and (d)−(f) the hybrid 'Mp-3'. cGISH signals using the genomic probe from *Ml* (red) with blocking DNA from *Ma* were detected in (g), (h) the hybrid 'Mp-3' and (j)−(l) *M. laevigata* 'L-1'. (i) The two-round cGISH showing overlapping signals from the *Ma* (green) and *Ml* (red) genomic probes with reciprocal blocking DNA. (m) Ideogram summarizing chromosome counts with different genomic probes' signals. White and red arrows point to the chromosomes with *Ma* and *Ml* signals, respectively. Blue arrows indicate the transmission of parental chromosome to the hybrid. Scale bars represent 5 μm.

**Figure 4:**
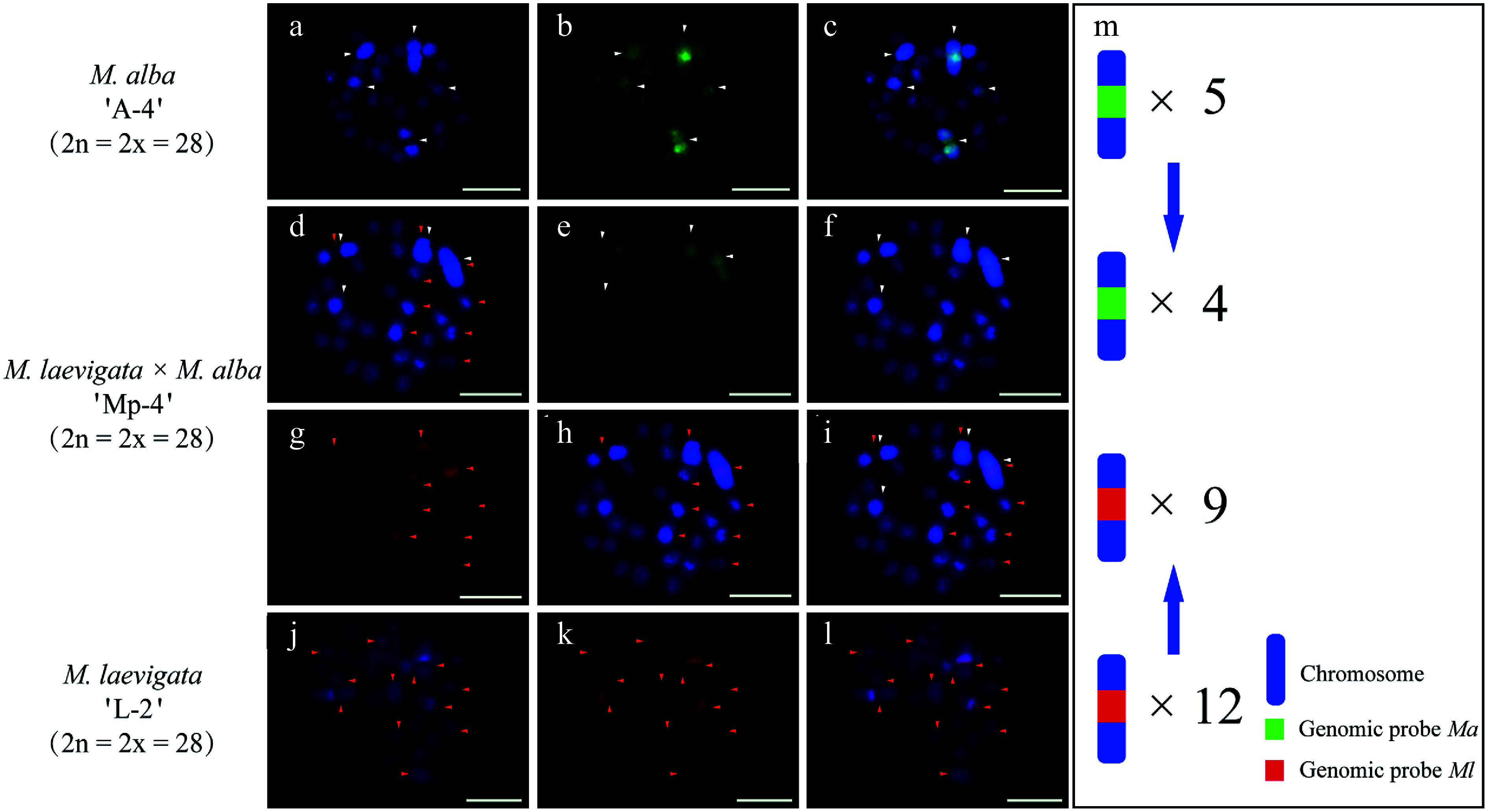
cGISH signal patterns in *M. alba* 'A-4', *M. laevigata* 'L-2', and their hybrid *M. laevigata × M. alba* 'Mp-4'. cGISH signals using the genomic probe from *Ma* (green) with blocking DNA from *M. laevigata* (*Ml*) were detected in (a)−(c) *M. alba* 'A-4' and (d)−(f) the hybrid 'Mp-4'. cGISH signals using the genomic probe from *Ml* (red) with blocking DNA from *Ma* were detected in (g), (h) the hybrid 'Mp-4' and (j)−(l) *M. laevigata* 'L-2'. (i) The two-round cGISH showing overlapping signals from the *Ma* (green) and *Ml* (red) genomic probes with reciprocal blocking DNA. (m) Ideogram summarizing chromosome counts with different genomic probes' signals. White and red arrows point to chromosomes with the *Ma* and *Ml* signals, respectively. Blue arrows indicate the transmission of parental chromosome to the hybrid. Scale bars represent 5 μm.

cGISH analysis of the *M. alba* 'A-3' × *M. laevigata* 'L-1' cross revealed distinct parental genomic distributions. When the *Ma* probe with *Ml* blocking DNA was applied, five hybridization signals were detected in the female parent (Fig. 3a−c). The *Ml* probe with *Ma* blocking DNA identified eight signals in the male parent (Fig. 3j−l). Their hybrid progeny, 'Mp-3', exhibited differential genomic incorporation, with five signals derived from each parent (Fig. 3d−i). A similar pattern was observed in the *M. wittiorum* 'L-2' × *M. alba* 'A-4' cross. The *Ml* probe with *Ma* blocking revealed 10 signals in the female parent (Fig. 4j−l), whereas the *Ma* probe with *Ml* blocking detected five signals in the male parent (Fig. 4a−c). In the resulting hybrid, 'Mp-4', four *Ma*-specific and nine *Ml*-specific signals were observed (Fig. 4d−i).

### GISH analysis of intersectional hybrids between *Morus* section *Laevigata* and section *Wittiorum*

Intersectional hybridization between *Morus* section *Laevigata* and section *Wittiorum* yielded two hybrid combinations: (1) *M.* *laevigata* 'L-2' × *M.* *wittiorum* 'W-3' and (2) *M.* *wittiorum* 'W-4' × *M.* *laevigata* 'L-1'. Genomic probes derived from *Ml* and *Mw* were used reciprocally as either hybridization probes or blocking DNA to analyze chromosomal compositions. Consistent with the abovementioned findings, the *Ml* and *Mw* genomic probes showed extensive signal overlap (Supplementary Fig. S4), necessitating cGISH with blocking DNA.

**Figure 5:**
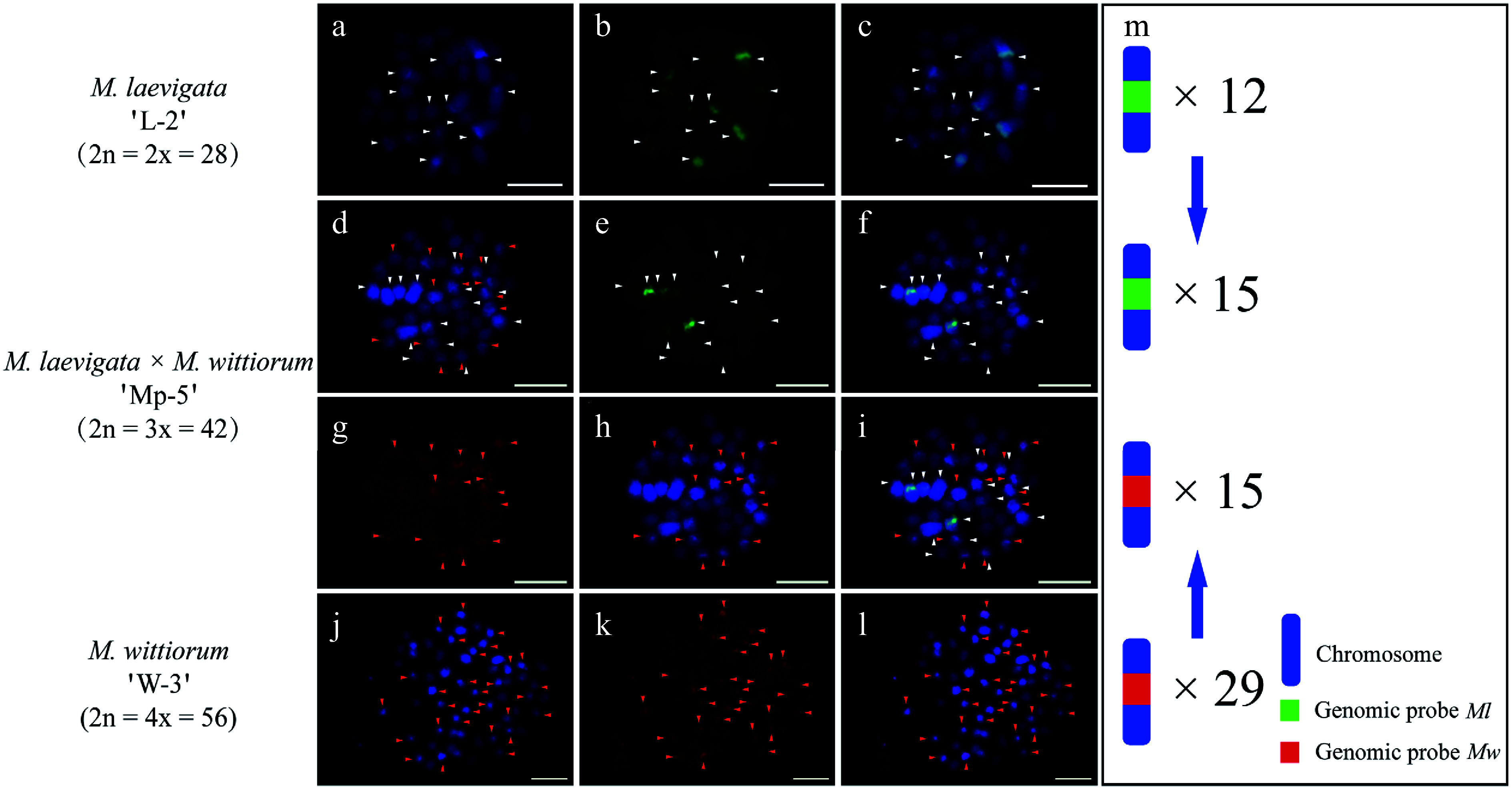
cGISH signal patterns in *M. laevigata* 'L-2', *M. wittiorum* 'W-3', and their hybrid *M. laevigata × M. wittiorum* 'Mp-5'. cGISH signals using the genomic probe from *Ml* (green) with blocking DNA from *Mw* were detected in (a)−(c) *M. laevigata* 'L-2' and (d)−(f) the hybrid 'Mp-5. cGISH signals using the genomic probe from *Mw* (red) with blocking DNA from *Ml* were detected in (g), (h) the hybrid 'Mp-5', and (j)−(l) *M. wittiorum* 'W-3'. (i) The two-round cGISH showing overlapping signals from the *Ml* (green) and *Mw* (red) genomic probes with reciprocal blocking DNA. (m) Ideogram summarizing chromosome counts with different genomic probes' signals. White and red arrows point to chromosomes with the *Ml* and *Mw* signals, respectively. Blue arrows indicate the transmission of parental chromosomes to the hybrid. Scale bars represent 5 μm.

**Figure 6:**
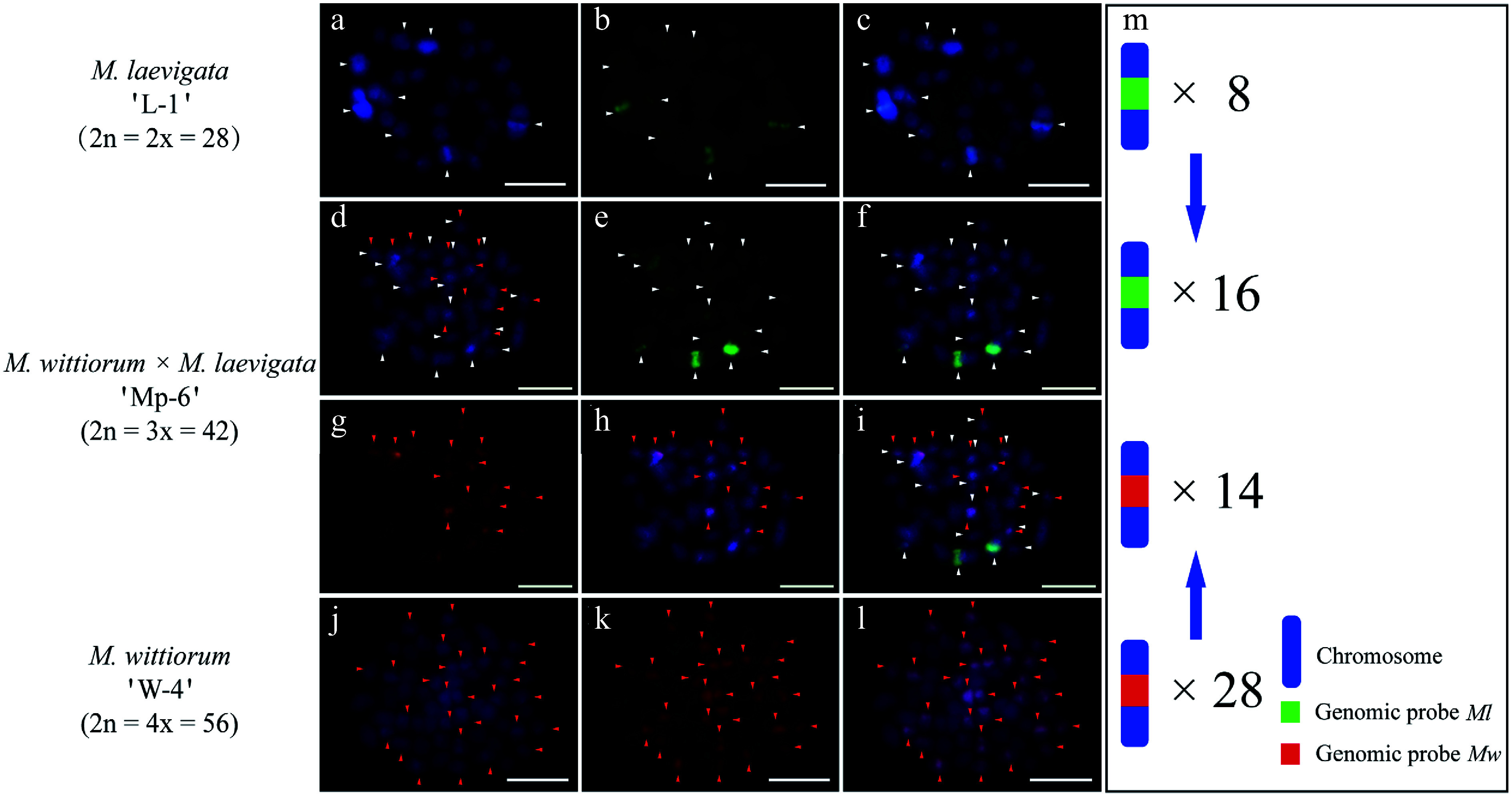
cGISH signal patterns in *M. laevigata* 'L-1', *M. wittiorum* 'W-4', and their hybrid *M. wittiorum × M. laevigata* 'Mp-6'. cGISH signals using the genomic probe from *Ml* (green) with blocking DNA from *Mw* were detected in (a)−(c) *M. laevigata* 'L-1' and (d)−(f) the hybrid 'Mp-6'. cGISH signals using the genomic probe from *Mw* (red) with blocking DNA from *Ml* were detected in (g), (h) the hybrid 'Mp-6', and (j)−(l) *M. wittiorum* 'W-4'. (i) The two-round cGISH showing overlapping signals from the *Ml* (green) and *Mw* (red) genomic probes with reciprocal blocking DNA. (m) Ideogram summarizing chromosome counts with different genomic probes' signals. White and red arrows point to the chromosomes with *Ml* and *Mw* signals, respectively. Blue arrows indicate the transmission of parental chromosomes to the hybrid. Scale bars represent 5 μm.

In the *M.* *laevigata* 'L-2' × *M.* *wittiorum* 'W-3' cross, the *Ml* probe with *Mw* blocking DNA identified 12 hybridization signals in the female parent (Fig. 5a−c). The *Mw* probe with *Ml* blocking DNA detected 29 signals in the male parent (Fig. 5j−l). Their hybrid progeny, 'Mp-5', exhibited 15 *Ml*-derived and 15 *Mw*-derived signals (Fig. 5d−i). In the case of *M. wittiorum* 'W-4' × *M. laevigata* 'L-1' exhibited parallel findings, as the *Mw* probe with *Ml* blocking revealed 28 signals in the female parent (Fig. 6j−l). The *Ml* probe with *Mw* blocking detected eight signals in the male parent (Fig. 6a−c). The hybrid 'Mp-6' demonstrated 16 *Ml*-specific and 14 *Mw*-specific signals (Fig. 6d−i).

**Figure 7:**
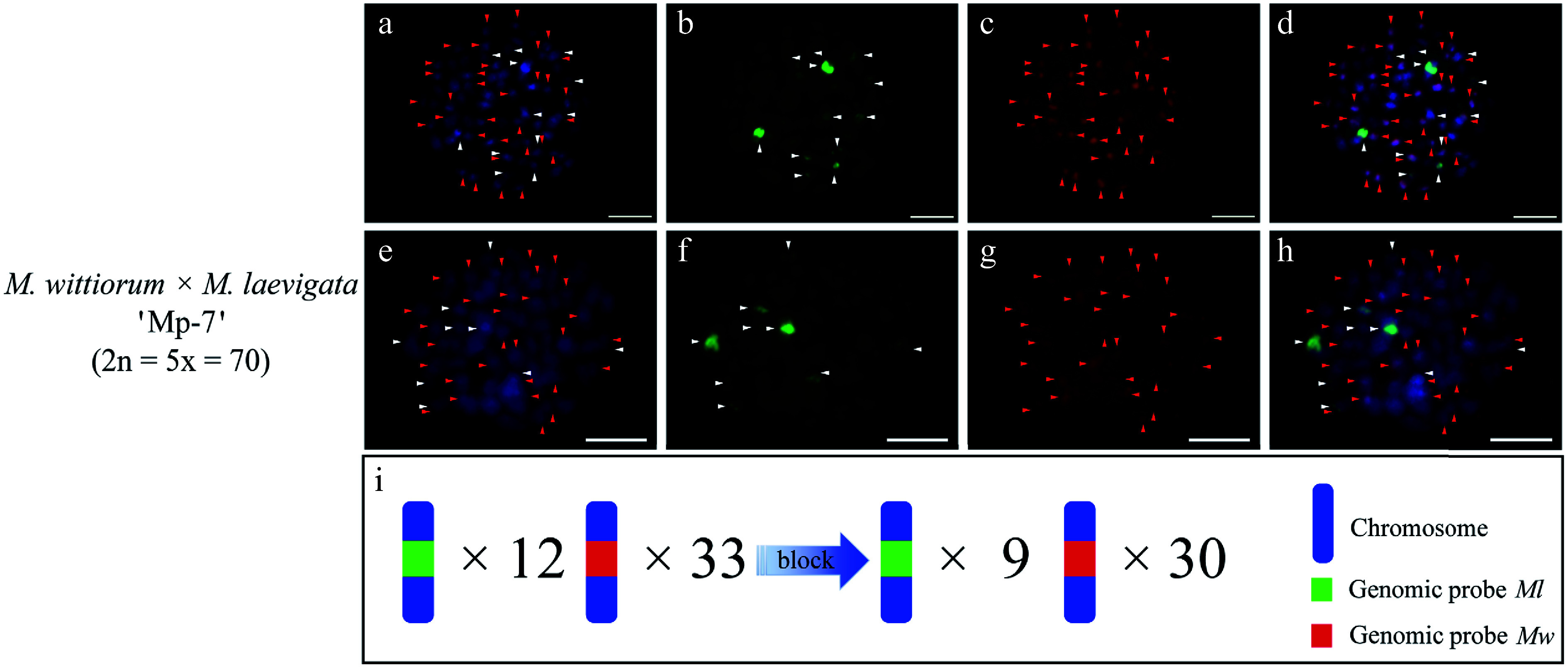
cGISH signal patterns in the hybrid 'Mp-7' from the cross *M. wittiorum* 'W-4' × *M. laevigata* 'L-1'. (a)−(d) Dual-color GISH signals using the genomic probes from *Ml* (green) and *Mw* (red) were detected in the hybrid 'Mp-7'. (e)−(h) The two-round cGISH signals using the genomic probes from *Ml* (green) and *Mw* (red) with reciprocal blocking DNA were detected in the hybrid 'Mp-7'. (i) Ideogram summarizing chromosome counts with different genomic probes' signals. White and red arrows point to chromosomes with the *Ml* and *Mw* signals, respectively. The blue gradient arrow indicates the changes in signal number after the application of blocking DNA. Scale bars represent 5 μm.

A pentaploid hybrid, designated 'Mp-7', was identified in the cross of *M.* *wittiorum* 'W-4' × *M.* *laevigata* 'L-1'. The genomic probes of *Ml* and *Mw* (no blocking DNA) hybridized to nearly all chromosomes (Fig. 7a−d). The *Ml* probe with *Mw* blocking DNA detected 9 clear signals, whereas the *Mw* probe with *Ml* blocker identified 30 signals in 'Mp-7' (Fig. 7e−h).

### GISH analysis of putative wild hybrids

**Figure 8:**
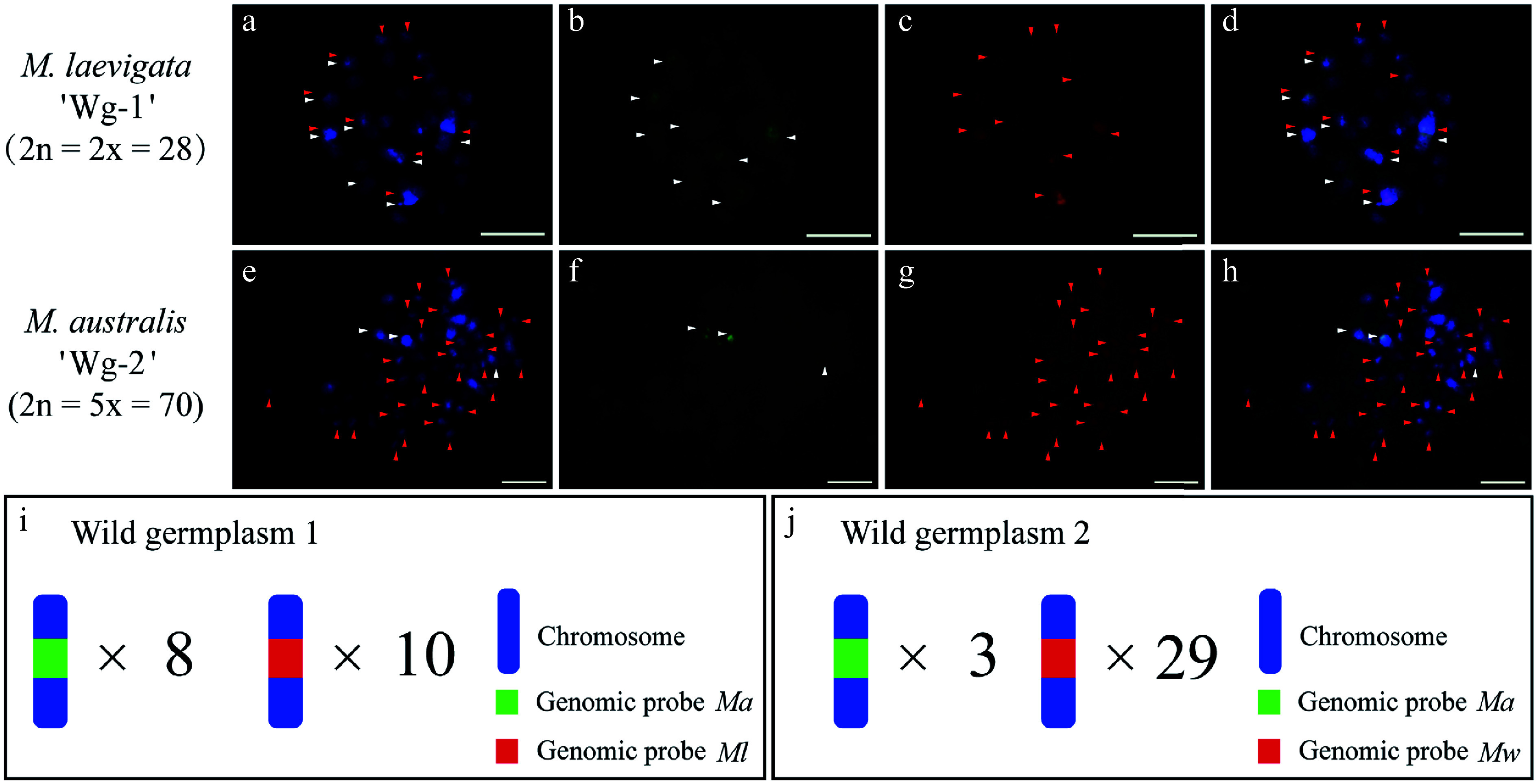
cGISH signal patterns in the wild accessions *M. laevigata* 'Wg-1' and *M. australis* 'Wg-2'. (a)−(d) The two-round cGISH signals using genomic probes from *Ma* (green) and *Ml* (red) with reciprocal blocking DNA were detected in *M. laevigata* 'Wg-1'. (e)−(h) Corresponding two-round cGISH signals using genomic probes from *Ma* (green) and *Mw* (red) with reciprocal blocking DNA were detected in *M. australis* 'Wg-2'. (i), (j) Ideograms summarizing chromosome counts with different genomic probes' signals. White arrows point to chromosomes with *Ma* signals, whereas red arrows point to chromosomes with (a)−(d) *Ml* signals or (e)−(h) *Mw* signals. Scale bars represent 5 μm.

On the basis of prior work demonstrating the utility of the *Ma* and *Mw* genomic probes for rapid mulberry genotyping^[20]^, we used these two probes to analyze the wild accessions *M. laevigata* 'Wg-1' and *M. australis* 'Wg-2' (Supplementary Fig. S5). The *Ma* probe hybridized to five chromosomal loci, whereas the *Mw* probe detected four clear signals in *M. laevigata* 'Wg-1' (Supplementary Fig. S5a1−a4). In *M. australis* 'Wg-2', the *Ma* probe marked chromosome 1 and chromosome 2, whereas *Mw* detected 31 signals (Supplementary Fig. S5b1−b4). According to the established cytotaxonomic criteria for mulberry^[20]^, this pattern suggested that 'Wg-L1' is a hybrid between sections *Laevigata* and *Alba*, and 'Wg-2' is a hybrid between sections *Alba* and *Wittiorum*. cGISH validation with blocking DNA was performed. The *Ma* probe with *Ml* blocking DNA produced 8 signals in *M. laevigata* 'Wg-1', whereas the *Ml* probe with *Ma* blocking DNA yielded 10 signals (Fig. 8a−d). In *M. australis* 'Wg-2', the *Ma* probe with *Mw* blocking detected 3 signals, whereas the *Mw* probe with *Ma* blocker revealed 29 signals (Fig. 8e, f).

## Discussion

### GISH with blocking DNA identifies mulberry hybrids

Cytology-based early hybrid authentication has been widely adopted as an efficient and reliable method for identifying hybrids in various plant species, including *Lilium* and *Capsicum*^[45−47]^. However, GISH faces challenges in *Morus* species because of the highly similar chromosome size and the cross-hybridization of genomic probes, which generate nonspecific signals across different mulberry sections. This limitation has been successfully overcome in other crops, such as rice (*Oryza sativa*) and kiwifruit (*Actinidia chinensis*), through the incorporation of blocking DNA, which suppresses repetitive DNA interference^[48,49]^. In this study, cGISH with blocking DNA enabled precise chromosomal delineation in hybrids derived from six cross combinations. Hybrid status was confirmed through a comparative analysis of quantitative signals, where the progeny exhibited approximately half the number of cGISH signals relative to their parents. For instance, when probed with *Ma* (blocked with *Mw* DNA), the hybrid 'Mp-1' displayed two distinct signals, contrasting with the four signals observed in its female parent *M. alba* 'A-1'.

Early hybrid authentication at the seedling stage is crucial for minimizing resource expenditure on false hybrids^[50]^. Given the prolonged juvenile phase in perennial species like *Morus*, rapid detection significantly accelerates breeding programs^[51,52]^. Our results demonstrate that GISH is a robust tool for identifying intersectional mulberry hybrids, facilitating efficient mulberry breeding programs. Moreover, the successful development and precise identification of intersectional hybrids provide a clear direction for advancing distant hybridization strategies in mulberry.

### Cytogenetic characterization of wild mulberry accessions using GISH with blocking DNA

As a wind-pollinated species with weak interspecific reproductive barriers, mulberry has generated numerous wild interspecific hybrids^[13,14,19,21,22]^. However, the parental origins and ploidy variability of these wild accessions remain poorly resolved^[20]^, significantly impeding their utilization in breeding. Although cGISH previously predicted 15 accessions as hybrids, their precise chromosomal composition was unresolved^[20]^. In this study, cGISH with blocking DNA outperformed dual-color GISH in clarifying the genomic constitution of wild mulberries, yielding unambiguous chromosomal compositions. Specifically, it was confirmed that *M. laevigata* 'Wg-1' (derived from sections *Laevigata* and *Alba*) and *M. australis* 'Wg-2' (from sections *Alba* and *Wittiorum*) are intersectional hybrids. *M. laevigata* 'Wg-1' exhibited a balanced *Laevigata*–*Alba* inheritance, whereas *M. australis* 'Wg-2' showed asymmetry, with *Wittiorum* being dominant.

Although dual-color GISH enables rapid hybrid screening, cGISH with blocking DNA facilitates precise chromosomal identification. Their complementary use provides a robust framework for dissecting the genetic complexity of wild mulberries. Furthermore, these validated wild hybrids represent valuable germplasm for trait introgression (e.g., via backcrossing), offering a faster alternative to conventional breeding by bypassing lengthy generation cycles.

### The genetic relationship of three mulberry sections revealed by GISH

Previous studies using cGISH with blocking DNA have identified distinct chromosomal signal patterns among *Morus* sections *Alba*, *Wittiorum*, and *Laevigata*^[20]^. In this study, we applied this approach to 10 mulberry accessions and seven intersectional hybrids derived from these sections, providing multidimensional insights into their genetic relationships and taxonomic boundaries. Variations in the probes' specificity and signal patterns were detected. For instance, the *Ml* probe produced differences in both signal numbers and intensities in *M. laevigata* 'L-2' under different blocking DNA conditions. Despite the presence of blocking DNA, the *Ml* probe consistently generated signals on chromosomes corresponding to its parental origin. This effect was further demonstrated in the hybrids 'Mp-3' and 'Mp-4', where overlapping two-round GISH signals were detected. Consequently, progeny derived from *laevigata* crosses exhibited a pronounced abundance of GISH signals, suggesting that *laevigata* genomes harbor diverse repeat sequences and share closer genetic affinity with section *Alba* than previously recognized. These results align with earlier observations in hybridization studies of *Erianthus rockii* × *N. porphyrocoma*^[53]^.

Furthermore, clear intersectional genetic divergence was observed. In the hybrids 'Mp-1' to 'Mp-6', differential signal intensities generated by different genomic probes confirmed substantial genomic differentiation among the three sections. Previously sequenced genomic data indicated a significant difference in the divergence time between *M. wittiorum* and *M. alba*^[54]^. Phylogenetic analyses based on plastomes and single-copy nuclear genes also demonstrated that the two species occupy distinct phylogenetic positions^[19]^. These results provide improved phylogenetic resolution for the three mulberry sections, refining their taxonomic boundaries and offering a framework for future studies on the dynamics of intersectional hybridization.

### Transmission patterns of parental chromosomes in mulberry hybrids

Although mulberry has traditionally been characterized by *n* + *n* inheritance, reports of abnormal meiotic behavior^[55,56]^ suggested potential deviations from this pattern. However, prior to this study, 2*n* gametes had never been documented in *Morus*. The standard *n* + *n* chromosomal inheritance pattern was consistently observed in the identified hybrids 'Mp-1' to 'Mp-6'. Here, we provide the first evidence of teh formation of 2*n* gametes in the female parent *M. wittiorum* 'W-4' (2*n* = 4*x* = 56), which generated the pentaploid hybrid 'Mp-7' (2*n* = 5*x* = 70). As is well known, there are three primary mechanisms underlie the formation of 2*n* gametes: the omission of one meiotic division, alterations in spindle morphology during meiotic division (e.g., parallel spindles), or defects in the cytokinesis of meiosis^[57−59]^. According to the genetic composition, 2*n* gametes are classified into two types: first division restitution (FDR) and second division restitution (SDR). FDR 2*n* gametes contain complete homologous chromosome pairs, comprising unreduced sets derived from both parents^[60]^. Consequently, SDR 2*n* gametes consist of sister chromatid pairs derived from a single parent, resulting in significantly lower heterozygosity compared with FDR gametes^[61]^. In this study, direct cytological evidence of abnormal female meiotic behavior in the maternal parent *M. wittiorum* 'W-4' was unobtainable because of the production of a 2*n* egg. Therefore, the specific mechanism responsible for the formation of 2*n* gametes in 'W-4' could not be determined. Further investigations into this mechanism will be conducted using cytogenetic analysis with other male mulberry accessions.

Moreover, a chromosome constitution similar to that of the pentaploid hybrid 'Mp-7' was detected in *M. australis* 'Wg-2', suggesting it may also be a pentaploid accession derived from the formation of 2*n* gametes. The findings indicate that ploidy variation in the genus *Morus* may be driven by the occurrence of 2*n* gametes, a phenomenon that is more prevalent in wild accessions than in cultivated accessions, which typically exhibit lower ploidy levels. Beyond their role in natural polyploidization, 2*n* gametes could have significant implications for breeding. They may stabilize the chromosomal composition in progeny, mitigate dosage effects, and overcome the reproductive barriers associated with odd-ploidy levels, thereby preserving breeding potential. This parallels their well-documented roles in polyploid breeding systems in species such as *Lilium, Eucommia,* and *Populus*^[62−66]^. Further research should focus on protocols for inducing 2*n* gametes and elucidating their underlying mechanisms in *Morus*. Such studies would advance our understanding of natural sexual polyploidization and support the development of targeted polyploid breeding strategies.

## Conclusions

In this study, cGISH with blocking DNA proved to be highly effective for early hybrid identification and precise analysis of the chromosomal composition across wild mulberry accessions. We present the first documented evidence of 2*n* gametes in *Morus*, showing that a maternal 2*n* egg from *M. wittiorum* 'W-4' (2*n* = 4*x* = 56) produced the pentaploid hybrid 'Mp-7' (2*n* = 5*x* = 70). These results establish GISH as a robust cytogenetic tool for authentication of species and hybrids in mulberry, while highlighting the critical role of 2*n* gametes in polyploidy-based breeding strategies. This work not only advances our understanding of natural polyploidization mechanisms in mulberry but also introduces innovative approaches for accelerating the genetic improvement of this agronomically significant species. Future studies should optimize GISH-based screening to improve breeding efficiency and explore the molecular pathways underlying the formation of 2*n* gametes.

## SUPPLEMENTARY DATA

Supplementary data to this article can be found online.

## Data Availability

The plant materials reported in the manuscript are included in Supplementary Tables S1 and S2 and are freely available to all the readers upon reasonable request.
