# Supplementary material for: Precise identification of intersectional hybrids in Morus using genomic in situ hybridization (GISH)
Source: For Res (Fayettev). 2026 Apr 3;6:e010. doi: 10.48130/forres-0026-0009 (PMC13191441; doi:10.48130/forres-0026-0009)
Supplement: Supplementary file 1 — Supplementary data to this article can be found online. [file FR-2026-6-009-S1.zip › 10.48130_forres-0026-0009-Suppl-FigureS3.pdf]

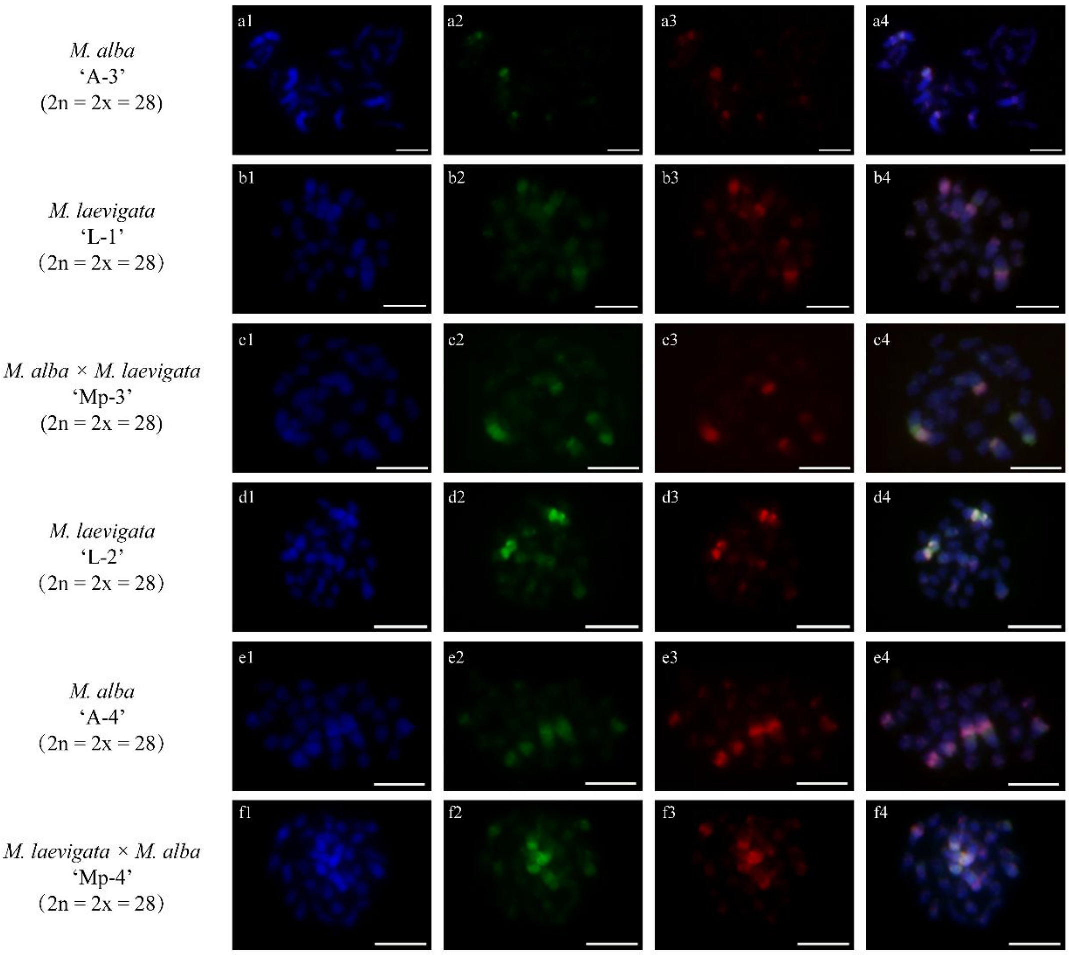

Fig. S3. cGISH signal patterns in *M. alba* 'A-3', *M. laevigata* 'L-1', their hybrid *M. alba* × *M. laevigata* 'Mp-3', *M. laevigata* 'L-2', *M. alba* 'A-4', and their hybrid *M. laevigata* × *M. alba* 'Mp-4'. Dual-color GISH signals of genomic probes of *Ma* (green) and *Ml* (red) in these mulberry accessions. **a1-4**: *M. alba* 'A-3', **b1-4**: *M. laevigata* 'L-1', **c1-4**: *M. alba* × *M. laevigata* 'Mp-3', **d1-4**: *M. laevigata* 'L-2', **e1-4**: *M. alba* 'A-4', **f1-4**: *M. laevigata* × *M. alba* 'Mp-4'. Scale bars represent 5 μm.
