# Supplementary material for: Precise identification of intersectional hybrids in Morus using genomic in situ hybridization (GISH)
Source: For Res (Fayettev). 2026 Apr 3;6:e010. doi: 10.48130/forres-0026-0009 (PMC13191441; doi:10.48130/forres-0026-0009)
Supplement: Supplementary file 1 — Supplementary data to this article can be found online. [file FR-2026-6-009-S1.zip › 10.48130_forres-0026-0009-Suppl-FigureS4.pdf]

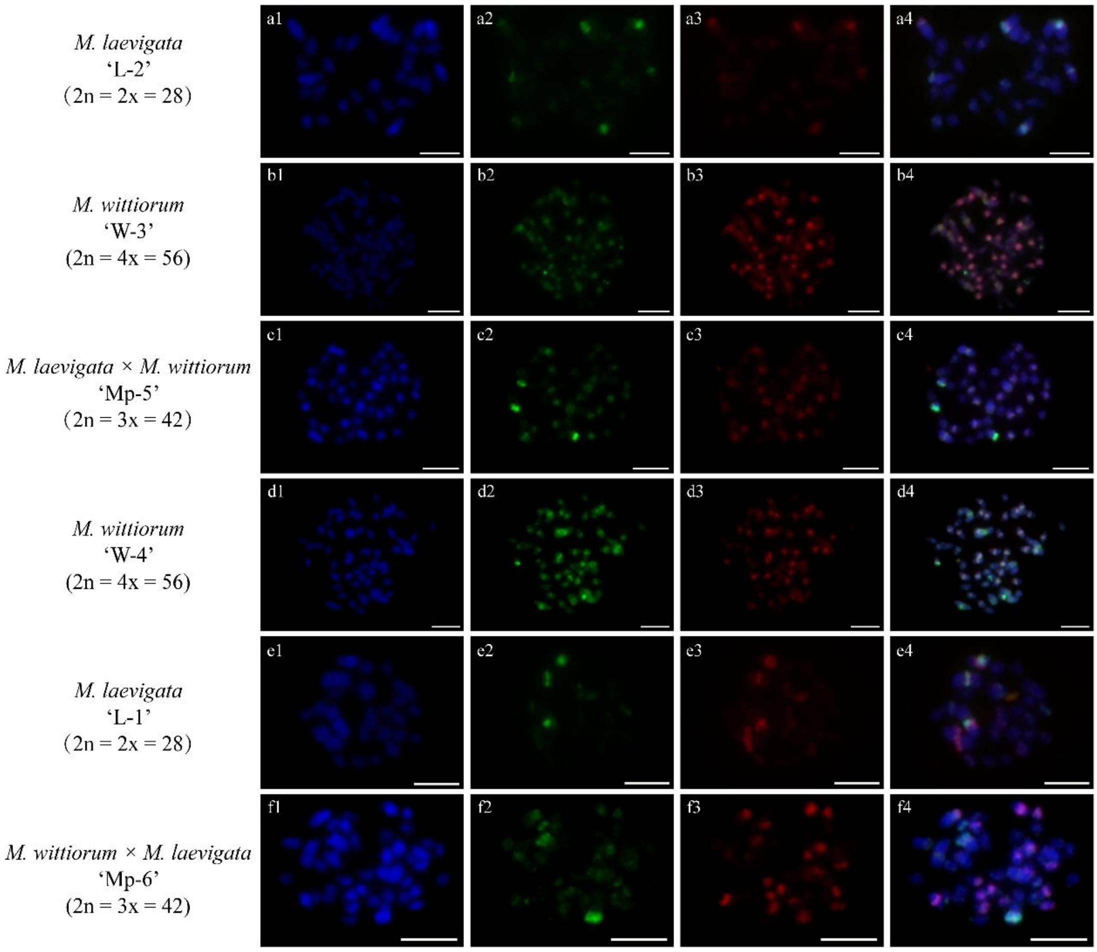

Fig. S4. cGISH signal patterns in *M. laevigata* 'L-2', *M. wittiorum* 'W-3', their hybrid *M. laevigata* × *M. wittiorum* 'Mp-5', *M. wittiorum* 'W-4', *M. laevigata* 'L-1', and their hybrid *M. wittiorum* × *M. laevigata* 'Mp-6'. Dual-color GISH signals of genomic probes of *Ml* (green) and *Mw*(red) in these mulberry accessions. **a1-4**: *M. laevigata* 'L-2', **b1-4**: *M. wittiorum* 'W-3', **c1-4**: *M. laevigata* × *M. wittiorum* 'Mp-5', **d1-4**: *M. wittiorum* 'W-4', **e1-4**: *M. laevigata* 'L-1', **f1-4**: *M. wittiorum* × *M. laevigata* 'Mp-6'. Scale bars represent 5 μm.
