# Supplementary material for: Precise identification of intersectional hybrids in Morus using genomic in situ hybridization (GISH)
Source: For Res (Fayettev). 2026 Apr 3;6:e010. doi: 10.48130/forres-0026-0009 (PMC13191441; doi:10.48130/forres-0026-0009)
Supplement: Supplementary file 1 — Supplementary data to this article can be found online. [file FR-2026-6-009-S1.zip › 10.48130_forres-0026-0009-Suppl-FigureS5.pdf]

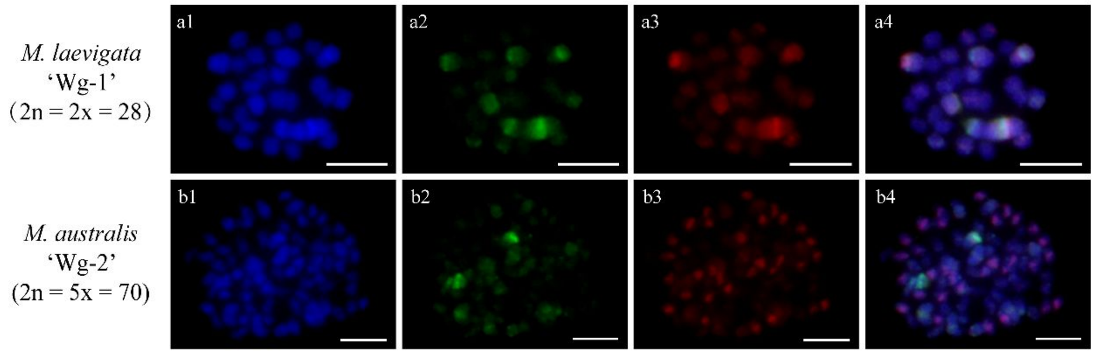

Fig. S5. cGISH signal patterns in *M. laevigata* 'Wg-1' and *M. australis* 'Wg-2'. Dual-color GISH signals of genomic probes of *Ma* (green) and *Mw*(red) in these mulberry accessions. **a1-4**: *M. laevigata* 'Wg-1', **b1-4**: *M. australis* 'Wg-2'. Scale bars represent 5 μm.
