# Supplementary material for: Precise identification of intersectional hybrids in Morus using genomic in situ hybridization (GISH)
Source: For Res (Fayettev). 2026 Apr 3;6:e010. doi: 10.48130/forres-0026-0009 (PMC13191441; doi:10.48130/forres-0026-0009)
Supplement: Supplementary file 1 — Supplementary data to this article can be found online. [file FR-2026-6-009-S1.zip › 10.48130_forres-0026-0009-Suppl-TableS1.pdf]

Supplementary Table 1. List of the mulberry accessions used in this study

No.	Code	Accession Name	Species Name	Mulberry section	Location (country)	Location (province)	Location (county)	Chromosome number	Ploidy Level	Means of Propagation
1	*Ma*	Heyebai	*M. multicaulis*	section *Alba*	China	Zhejiang	Haining	28	2	grafting
2	*Ml*	Menghai No. 2	*M. laevigata*	section *Laevigata*	China	Yunnan	Menghai	28	2	grafting
3	*Mw*	Ailaoshan No. 9	*M. wittiorum*	section *Wittiorum*	China	Yunnan	Pu'er	56	4	grafting
4	*M. alba* 'A-1'	Baiyuwang	*M. alba*	section *Alba*	China	Shanxi	Yangling	56	4	grafting
5	*M. alba* 'A-2'	Guanjingtai No. 3	*M. mongolica*	section *Alba*	China	Yunnan	Longling	28	2	grafting
6	*M. alba* 'A-3'	Jisang	*M. australis*	section *Alba*	China	Chongqing	Beibei	28	2	grafting
7	*M. alba* 'A-4'	Shengnan	*M. alba*	section *Alba*	China	Japan	N/A	28	2	grafting
8	*M. laevigata* 'L-1'	Jinghong No. 1	*M. laevigata*	section *Laevigata*	China	Yunnan	Jinghong	28	2	grafting
9	*M. laevigata* 'L-2'	Yun6muben	*M. laevigata*	section *Laevigata*	China	Yunnan	Mengzi	28	2	grafting
10	*M. wittiorum* 'W-1'	Sangshuwang No. 2	*M. wittiorum*	section *Wittiorum*	China	Yunnan	Baoshan	56	4	grafting
11	*M. wittiorum* 'W-2'	Ailaoshan No. 3	*M. wittiorum*	section *Wittiorum*	China	Yunnan	Puer	56	4	grafting
12	*M. wittiorum* 'W-3'	Sangshuwang	*M. wittiorum*	section *Wittiorum*	China	Yunnan	Baoshan	56	4	grafting
13	*M. wittiorum* 'W-4'	Ailaoshan No. 2	*M. wittiorum*	section *Wittiorum*	China	Yunnan	Puer	56	4	grafting
14	*M. laevigata* 'Wg-1'	Natural-pollinated progeny 1 of Yun6muben	*M. laevigata*	section *Laevigata*×section *Alba*	China	Chongqing	Beibei	28	2	seedings
15	*M. australis* 'Wg-2'	Longling No. 2	*M. australis*	section *Alba*×section *Wittiorum*	China	Yunnan	Baoshan	70	5	grafting
