# Supplementary material for: Precise identification of intersectional hybrids in Morus using genomic in situ hybridization (GISH)
Source: For Res (Fayettev). 2026 Apr 3;6:e010. doi: 10.48130/forres-0026-0009 (PMC13191441; doi:10.48130/forres-0026-0009)
Supplement: Supplementary file 1 — Supplementary data to this article can be found online. [file FR-2026-6-009-S1.zip › 10.48130_forres-0026-0009-Suppl-TableS2.pdf]

Supplementary Table 2. List of seven mulberry accessions from six hybrid combinations

No.	Code	Supposed Parents (Mo × Fa)	Chromosome number	Ploidy Level
1	Mp-1	*M. alba*'Baiyuwang' × *M. wittiorum* 'Sangshuwang No. 2'	56	4
2	Mp-2	*M. wittiorum* 'Ailaoshan No. 3' × *M. mongolica* 'Guanjingtai No. 3'	42	3
3	Mp-3	*M. australis* 'Jisang' × *M. laevigata* 'Jinghong No. 1'	28	2
4	Mp-4	*M. laevigata* 'Yun6muben' × *M. alba* 'Shengnan'	28	2
5	Mp-5	*M. laevigata* 'Yun6muben' × *M. wittiorum* 'Sangshuwang'	42	3
6	Mp-6	*M. wittiorum* 'Ailaoshan No. 2' × *M. laevigata* 'Jinghong No. 1'	42	3
7	Mp-7	*M. wittiorum* 'Ailaoshan No. 2' × *M. laevigata* 'Jinghong No. 1'	70	5
