# Supplementary material for: Precise identification of intersectional hybrids in Morus using genomic in situ hybridization (GISH)
Source: For Res (Fayettev). 2026 Apr 3;6:e010. doi: 10.48130/forres-0026-0009 (PMC13191441; doi:10.48130/forres-0026-0009)
Supplement: Supplementary file 1 — Supplementary data to this article can be found online. [file FR-2026-6-009-S1.zip › 10.48130_forres-0026-0009-Suppl-FigureS1.pdf]

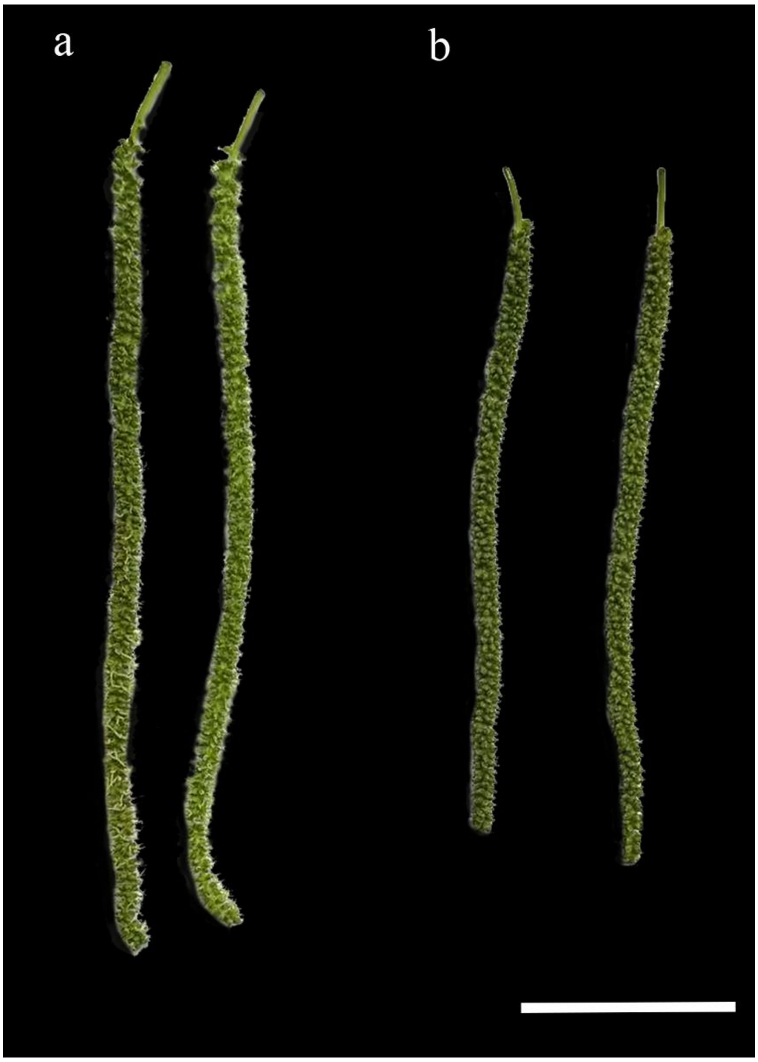

Fig. S1. Fruit morphological characteristics of *M. laevigata* and *M. wittiorum*. a: *M. laevigata* ‘Yun6muben’, **b**: *M. wittiorum* ‘Ailaoshan No. 9’. Scale bars represent 5cm.
